# *Read-SpaM*: assembly-free and alignment-free comparison of bacterial genomes with low sequencing coverage

**DOI:** 10.1186/s12859-019-3205-7

**Published:** 2019-12-17

**Authors:** Anna-Katharina Lau, Svenja Dörrer, Chris-André Leimeister, Christoph Bleidorn, Burkhard Morgenstern

**Affiliations:** 1 Universität Göttingen, Department of Bioinformatics, Goldschmidtstr. 1, 37073 Göttingen, Germany; 2 Universität Göttingen, Department of Animal Evolution and Biodiversity, Untere Karspüle 2, Göttingen, 37073 Germany

**Keywords:** Alignment-free, Phylogenomics, Unassembled reads

## Abstract

**Background:**

In many fields of biomedical research, it is important to estimate phylogenetic distances between taxa based on low-coverage sequencing reads. Major applications are, for example, phylogeny reconstruction, species identification from small sequencing samples, or bacterial strain typing in medical diagnostics.

**Results:**

We adapted our previously developed software program *Filtered Spaced-Word Matches (FSWM)* for alignment-free phylogeny reconstruction to take unassembled reads as input; we call this implementation *Read-SpaM*.

**Conclusions:**

Test runs on simulated reads from semi-artificial and real-world bacterial genomes show that our approach can estimate phylogenetic distances with high accuracy, even for large evolutionary distances and for very low sequencing coverage.

## Background

Phylogeny reconstruction is a basic task in biological sequence analysis [1]. Traditionally, phylogenetic trees of species are calculated from carefully selected sets of marker genes or proteins. With the huge amounts of sequencing data that are produced by novel sequencing technologies, genome-based phylogeny reconstruction or *phylogenomics* has become a standard approach [2, 3]. Here, the usual workflow is as follows: DNA sequencing produces a large number of reads, these reads are then assembled to obtain contigs or complete genomes. From the assembled sequences, orthologous genes are identified and multiple alignments of these genes are calculated. Finally, phylogeny-reconstruction methods such as *Maximum Likelihood* [4] are applied to these alignments to obtain a phylogenetic tree of the species under study. This procedure is time-consuming and error-prone, and it requires manual input from highly-specialized experts.

In recent years, a large number of alignment-free approaches to phylogeny reconstruction have been developed and applied, since these methods are much faster than traditional, alignment-based phylogenetic methods, see [5–8] for recent review papers and [9] for a systematic evaluation of alignment-free software tools. Most alignment-free approaches are based on *k*-mer statistics [10–16], but there are also approaches based on the *length of common substrings* [17–22], on word or spaced-word matches [11, 23–27] or on so-called *micro-alignments* [28–31]. As has been mentioned by various authors, an additional advantage of many alignment-free methods is that they can be applied not only to assembled genome sequences, but also to unassembled reads. This way, the time-consuming and unreliable procedure of genome-assembly can be skipped. Assembly-free approaches can be applied, in principle, to low-coverage sequencing data. While proper genome assembly requires a coverage of around 30 reads per position, assembly-free approaches have been shown to produce good results with far lower sequencing coverage. This makes the new approach of *genome skimming* [32–37] possible, where low-coverage sequencing data are used to identify species or bacterial strains, for example in biodiversity studies [37] or in clinical applications [38, 39].

Alignment-free methods, including *Co-phylog* [28], *Mash* [24], *Simka* [40], *AAF* [41] and *Skmer* [37], have been successfully applied to unassembled reads. *Co-phylog* estimates distances using so-called *micro alignments*. In benchmark studies, this program could produce trees of very high quality, provided the sequencing depth was 6*X* and higher. Similarly, the programs *Mash* and *Simka* work on complete genomes as well as on unassembled reads. The required sequencing depth for these programs is comparable to the depth required by *Co-phylog*. The program *AAF* has been especially developed to work on unassembled data, it filters single copy *k-mers* to balance sequencing errors. This program produces accurate results and requires a sequencing coverage of ≥5*X*.

In this paper, we introduce an alignment-free and assembly-free approach to estimate evolutionary distances, that is based on our previously introduced software *Filtered Spaced-Word Matches (FSWM)* [30]. *FSWM* is a fast performing program for phylogeny reconstruction. It is based on gap-free local *micro-alignments*, so-called *spaced-word matches*. Originally the program was developed to estimate distances between genome sequences; there is also an implementation of this approach called *Prot-SpaM* that can compare whole-proteome sequences to each other [31]. In the present study, we adapted *FSWM* to take unassembled sequencing reads as input. Our program can compare either a set of unassembled reads from one taxon to an assembled genome of another taxon or two sets of unassembled reads to each other, each set from one taxon. Using simulated reads, we show that this method can accurately calculate distances between a complete genome and a set of reads for coverages down to 2^−9^*X*. If two sets of reads are compared, the method still works for coverages down to to 2^−6^*X*.

The paper is organized as follows: In the next section, we shortly recapitulate how the program *FSWM* works, and we explain the modifications that we implemented to use unassembled reads as input data. In the subsequent section, the benchmark setup and evaluation procedure are described. Next, we report on our benchmark results, and in the “Discussion” section, our results are discussed and possible future applications are addressed.

## Estimating phylogenetic distances with *FSWM* and *Read-SpaM*

**Fig. 1:**
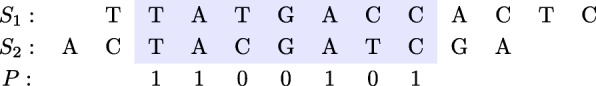
Spaced-word match. between two DNA sequences *S*_1_ and *S*_2_ with respect to a binary pattern *P*=1100101 of length *ℓ*=7, representing *match positions* (‘1’) and *don’t-care positions* (‘0’). The two segments have matching nucleotides at all *match positions* of *P* but may mismatch at the *don’t-care* positions

For our approach, we first need to specify a binary pattern *P* of representing *match positions* and *don’t-care positions* [42, 43]. Let *ℓ* be the length of the pattern *P*. A spaced-word match between two DNA sequences with respect to *P* is a pair of length- *ℓ* segments, one segment from each of the sequences, such that these segments have matching nucleotides at the *match positions* of *P*. Mismatches are allowed at the *don’t-care* positions, see Fig. 1 for an example. In other words, a spaced-word match is a gap-free local pairwise alignment of length *ℓ*, with matching nucleotides at the *match positions* of *P* and possible mismatches elsewhere.

Our previously published program *FSWM* [30] estimates the *Jukes-Cantor* distance [44] between two DNA sequences as follows: first all spaced-word matches between the sequences are identified with respect to a pre-defined pattern *P*. In order to distinguish spaced-word matches representing true homologies from background spaced-word matches, a score is calculated for each spaced-word match by summing up nucleotide substitution scores for the pairs of nucleotides that are aligned at the *don’t-care* positions of *P*. Here we use a substitution matrix that has been proposed by Chiaromonte et al. [45]. Spaced-word matches with scores below some threshold value *T* are discarded. The remaining (‘filtered’) spaced-word matches are then used to estimate the distance between the sequences: The average number of mismatches per position is calculated for all *don’t-care* positions of the non-discarded spaced-word matches, and the *Jukes-Cantor* correction is used to estimate the number of substitutions per position since the sequences have evolved from their last common ancestor.

In the present study, we adapted *FSWM* to compare unassembled reads to each other or to assembled genomes. We call this implementation *Read-SpaM* (for *Read-based* *Spaced-Word* *Matches*). There are two ways in which *Read-SpaM* can be used: (1) a set of unassembled sequencing reads from one taxon can be compared to a partially or fully assembled genome from another taxon; (2) a set of reads from one taxon can be compared to a set of reads from a second taxon. In both cases, all spaced-word matches between the reads and the genome or between the reads from the first taxon and the reads from the second taxon are identified and used to estimate the *Jukes-Cantor* distance between the two taxa as outlined above.

To run on short sequencing reads, we modified the length of the underlying binary patterns used in the program. While the original *FSWM* uses by default a pattern length of 112 and 12 *match positions*, *Read-SpaM* uses by default patterns of length 72, also with 12 *match positions*, i.e. with 60 *don’t-care positions*. A suitable pattern was calculated with the software *Rasbhari* [46]. As in the original *FSWM*, we are using the nucleotide substitution matrix by Chiaromonte et al. [45] and a threshold value of *T*=0. That is, we discard all spaced-word matches for which the sum of the scores of the aligned nucleotides at the 60 *don’t-care* positions is smaller than 0. *Read-SpaM* takes *FASTA*-formatted sequence files as input, one file per input taxon.

If we want to estimate phylogenetic distances from unassembled reads as described above, we have to take sequencing errors into account. Studies have shown that *Illumina* sequencing systems have error rates of 0.24±0.06*%* per position [47]. Our software corrects for these errors before it calculates distances between a set of reads and a genomes, or between two different sets of reads.

## Benchmark Setup

To evaluate *Read-SpaM*, we used simulated reads for three types of test scenarios: (1) Pairs of one real and one semi-artificial genome, respectively, with known phylogenetic distances, to compare estimated distances to real distances for a large range of distance values, (2) pairs of real genomes from different strains of *E. coli* and (3) sets of 17 different bacterial taxa, where we used full genome sequences from 16 taxa and unassembled reads from a 17th taxon. In (1) and (2), we estimated phylogenetic distances with *Read-SpaM* and, as a comparison, with the program *Mash* [24], and we compared the obtained distances to the reference distances. *Mash* was run with default parameter values. In (3), we reconstructed phylogenetic trees based on the *Read-SpaM* distances and compared them to trusted reference trees.

In all three cases, we simulated sequencing reads with the software tool *ART* [48]. *ART* can simulate next-generation sequencing reads from the three main commercial sequencing platforms with technology-specific read error models, including *Illumina*. In our test runs, we used the *Illumina HiSeq 2500* sequencing system, as it is still a widely used system in the field. The length of a single simulated read in our study is 150 bp, since this is the standard length of reads produced by *Illumina HiSeq 2500*.

Further settings were chosen as follows: The highest sequencing coverage in our study is 1*X*, and we reduced the coverage in our test runs down to 2^−9^*X*. This way, we could identify the minimum sequencing coverage for which one can still obtain reasonable distance estimates, for a given evolutionary distance. *ART* randomly selects positions of the genome sequences from which reads are simulated. Consequently, the generated sets of reads can vary considerably. We therefore generated 10 sets of simulated reads for each pair of genomes and level of sequencing depth, and we report the average and standard deviations of the estimated distances over the 10 sets of reads.

### Semi-artificial pairs of genomes

In our first test scenario, semi-artificial genome pairs were generated as follows: We used one real genome from *E. coli* and then generated a second, semi-artificial genome by simulating nucleotide-acid substitutions, as well as insertions and deletions (indels). Indels were generated randomly with a probability of 1% at every position in the genome; the length of each indel was chosen randomly between 1 and 100, with a uniform length distribution. Various substitution probabilities were used to generate sequence pairs. We did a first series of test runs with evolutionary distances between 0 and 1 substitutions per position, and a second series with distances between 0 and 0.1 substitutions per position.

### Real-world genome pairs

In addition to these test runs on semi-artificial genome sequences, we used pairs of real genomes from different strains of *E. coli*, with evolutionary distances between 0.003 and 0.023 substitutions per position. We compared the distances obtained with *Read-SpaM* and *Mash* based on unassembled reads to the distances calculated by *FSWM* from the corresponding assembled genomes. Again, we first compared one assembled genome to a set of simulated reads from the respective second genome; then we compared sets of unassembled reads from both genomes to each other.

We should mention that there is a certain bias in the distances estimated by *FSWM* if real-world genomes are compared. As explained in [30], *FSWM* considers *all spaced-word matches* between two compared genomes w.r.t. a given binary pattern, i.e. all local-gapfree alignments with matching nucleotides at certain pre-defined positions, and with scores above some threshold. Distances are then estimated from the number of mismatches in these gap-free *micro-alignments*. Since *FSWM* will find more spaced-word matches per position in regions of high sequence similarity than in regions of lower similarity, the overall similarity between the sequences is *over-estimated* by the program, i.e. the estimated distances are too small.

To mitigate this bias, one can split the first genome into fragments and compare each fragment individually to the complete second genome. The overall distance between the genomes is then estimated as the *average* distance over all fragments. In our study, we used both distances as reference, the uncorrected distance estimated by *FSWM* as well as the distance that is based on fragmenting one of the compared genomes. For the ‘fragmented’ version of *FSWM*, we split one of the two compared genomes into 2000 fragments of equal length. Neighboring fragments have an overlap of *ℓ*−1, where *ℓ* is the length of the binary pattern, to ensure that at each position of the fragmented genome, the *ℓ*-mer at this position is contained in exactly one of the fragments.

### Wolbachia Phylogeny

As a third set of test cases, we used genome sequences of 13 *Wolbachia* strains from the lineages (“supergroups”) A - D; plus 4 strains of closely related Alphaproteobacteria that we used as an outgroup. *Wolbachia* belong to the Alphaproteobacteria and are intracellular endosymbionts of arthropods and nematodes, see [49] for classification of *Wolbachia*. As a reference tree, we used a tree published by [50]. We generated four sequence data sets, each set consisting of 12 assembled *Wolbachia* genome sequences, a set of unassembled reads with coverage 1*X* from the respective 13th *Wolbachia* strain, and the 4 assembled genomes sequences from the outgroup taxa. We then applied *Read-SpaM* and *FSWM* to estimate phylogenetic distances within each data set, and calculated trees from these distance matrices with the *Neighbor-Joining* [51] implementation from the *PHYLIP* package [52].

## Results

**Fig. 2:**
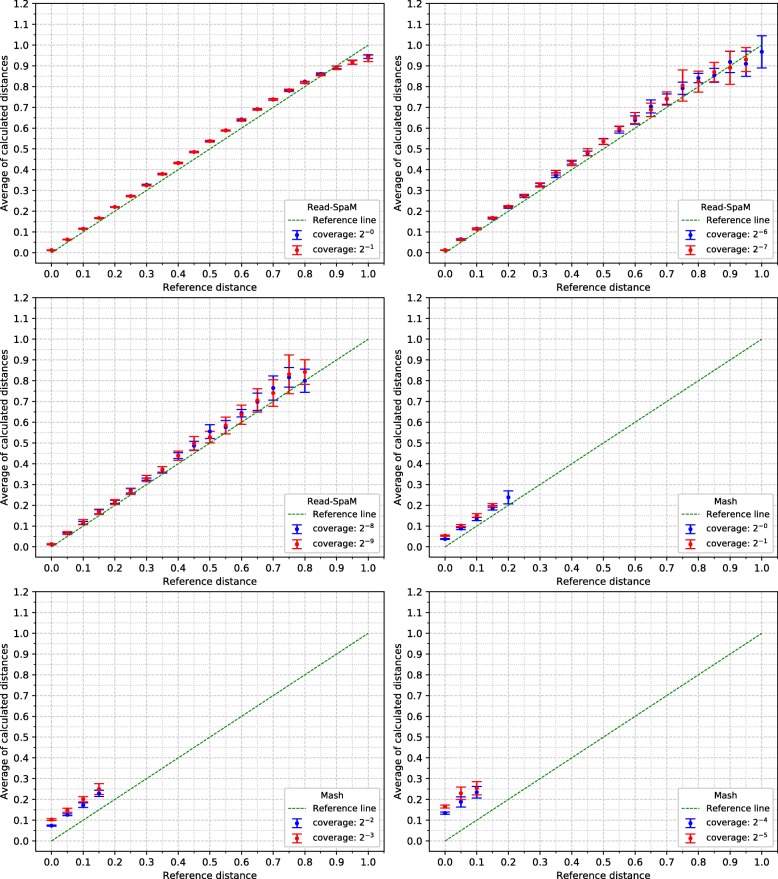
Estimated vs. reference distances, reads against genomes, for large distance values up to one substitution per sequence position. Phylogenetic distances between semi-artificial assembled genomes and unassembled reads (see main text), estimated by *Read-SpaM* and *Mash*. Estimated distances are plotted against the real distances for different values of sequencing coverage between 1*X* and 2^−9^*X*. Error bars represent standard deviations

**Fig. 3:**
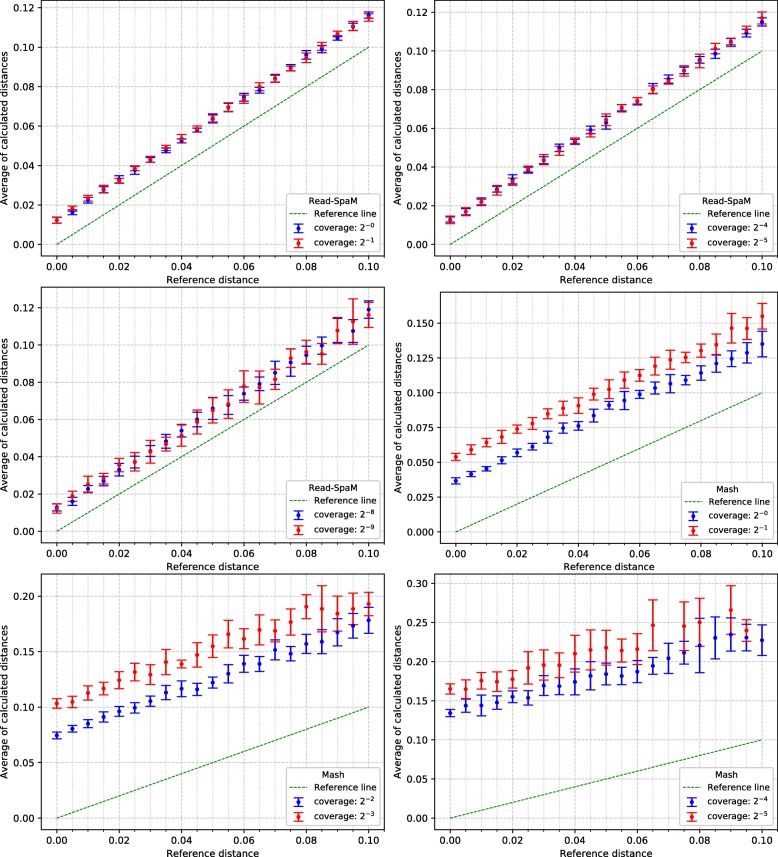
Estimated vs. reference distances, reads against genomes, for small distance values. up to 0.1 substitutions per sequence position. Notation as in Fig. 2

**Fig. 4:**
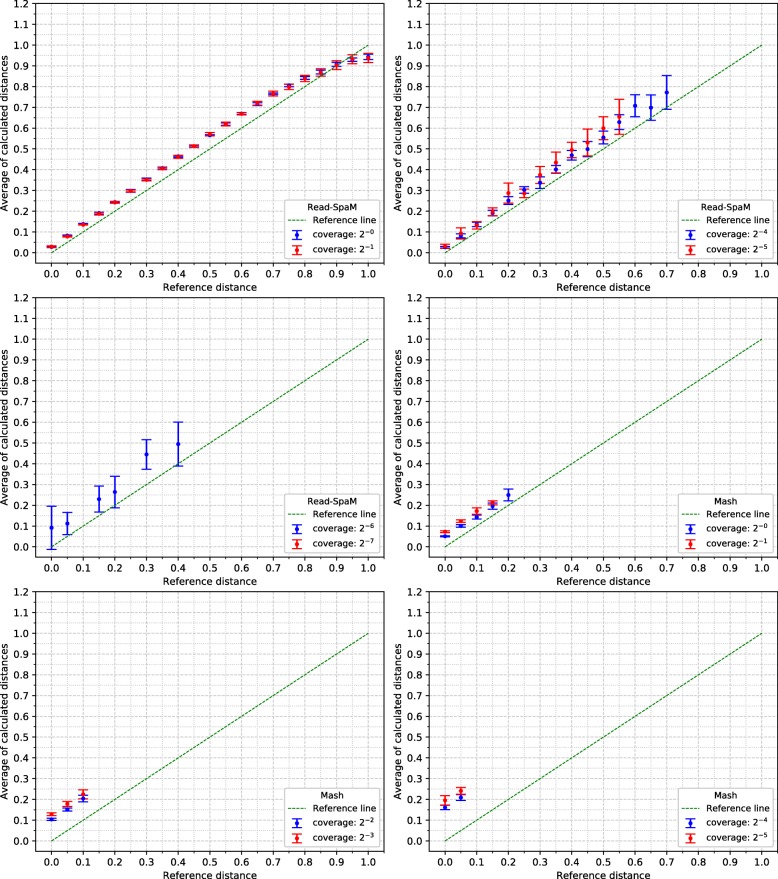
Estimated vs. reference distances, reads against reads, for large distance values up to 1 substitution per sequence position. Notation as in Fig. 2

**Fig. 5:**
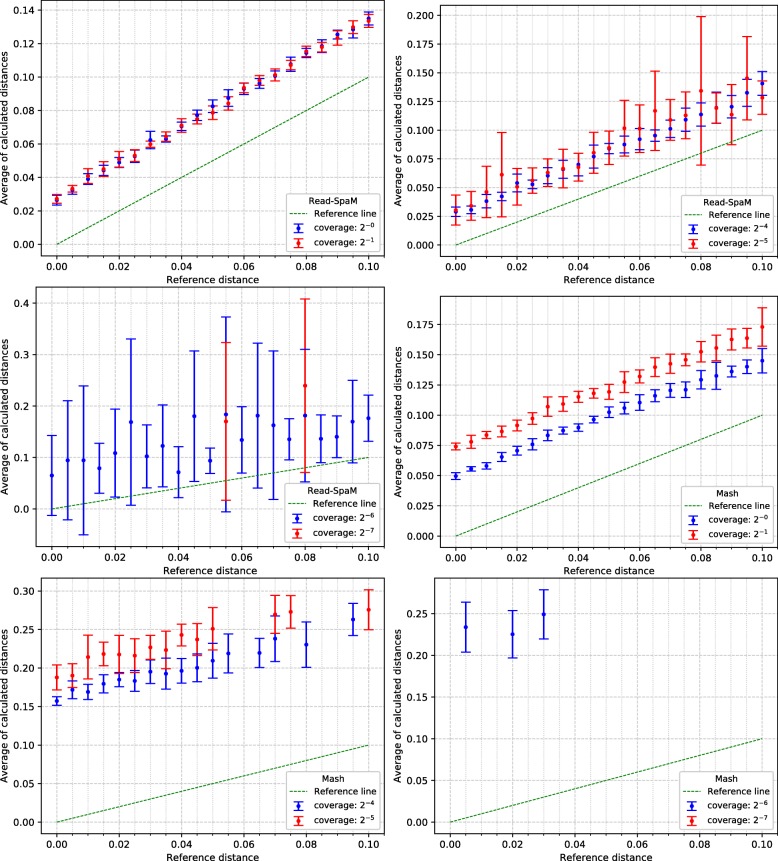
Estimated vs. reference distances, reads against reads, for small distance values. up to 0.1 substitutions per position. Notation as in Figure 2

**Fig. 6:**
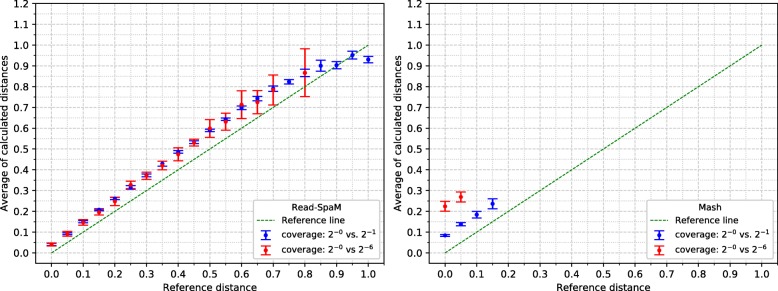
Estimated vs. reference distances, reads against reads as in Fig. 4, but with different sequencing coverage in the compared sequences. Notation as in Fig. 2

For the semi-artificial sequence pairs – each pair consisting of one real genome and one artificial genome with known distance to the real genome –, we first applied *Read-SpaM* and *Mash* to estimate distances between one assembled genome and unassembled reads from the second genome. As mentioned above, for each distance and level of sequencing coverage, we generated 10 sets of reads. In Fig. 2, the average and standard deviation of the 10 obtained distance values is plotted against the real distance of the two genomes for distance values between 0 and 1 substitutions per position. In addition, we did the same experiments for simulated sequences with smaller distances. Figure 3 shows the results for distances between 0 and 0.1 substitutions per position. Standard deviations are represented as error bars in the figures. Next, we used the same semi-artificial genome pairs as above, but we generated simulated reads for *both* genome sequences from each pair and compared them to each other. The results for the comparison of unassembled reads from one genome against unassembled reads from a second genome are shown in Fig. 4 and Fig. 5. In these test runs, we used the same sequencing coverage for both compared genomes. We obtained similar results when we compared sets of reads with different sequencing coverage for both compared genomes; two examples are shown in Fig. 6.

*Read-SpaM* and *Mash* are based on spaced-word or *k*-mer matches. Thus, they can produce meaningful results only if such matches can be found, given the underlying binary pattern *P* or word length *k*, respectively. If the sequencing coverage is too low and/or the evolutionary distance between the compared sequences is too large, it happens that no spaced-word or *k*-mer matches are found, and the distance between the sequences cannot be estimated. As mentioned, we generated 10 sets of reads for each genome pair and level of sequencing coverage in our test runs and used the average of the estimated distance values over the 10 test runs. In Fig. 2 to Fig. 5, we report results only for those test cases, in which the evaluated program was able to estimate distances for *all* of the 10 sets of simulated reads. In Fig. 2, for example, this was the case for all distances up to 1 substitution per position, for a sequencing coverage from 1*X* down to 2^−6^*X*. With a coverage of 2^−7^*X*, only distances up to 0.95 could be estimated for all 10 sets of reads, while for a coverage of 2^−8^*X* and 2^−9^*X*, this was only possible for distances up to 0.8 substitutions per positions. For larger distances, no output was produced for at least one of the 10 sets of simulated reads, so no results are reported for these parameters in Fig. 2. *Mash*, by contrast, produced results for all 10 data sets only for distances up to 0.2 when the coverage was 1. For a coverage between 2^−1^*X* and 2^−3^*X*, distances for all 10 data sets could only be calculated for sequences with a distance of up to 0.15. For a coverage of 2^−6^*X* and lower, *Mash* did not produce reliable estimates for any of the strictly positive distance values that we tested.

**Fig. 7:**
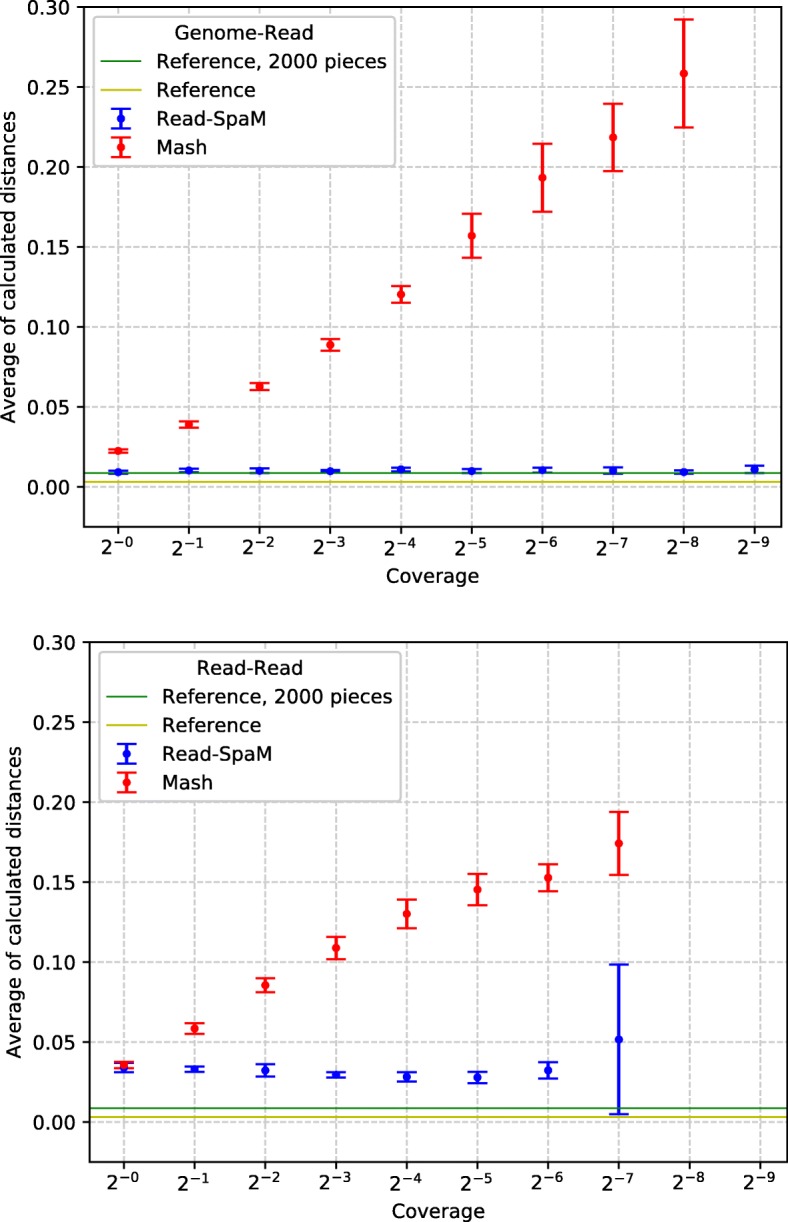
Distances between *E.coli* strains B4Sb227 and BW2952, estimated by *Read-SpaM* and *Mash* using simulated reads from one genome and the assembled second genome (top) and simulated reads from both genomes (bottom) for different levels of sequencing coverage. Horizontal lines are reference distances, estimated by *FSWM* from the assembled full genomes and using fragmented genomes (see main text)

**Fig. 8:**
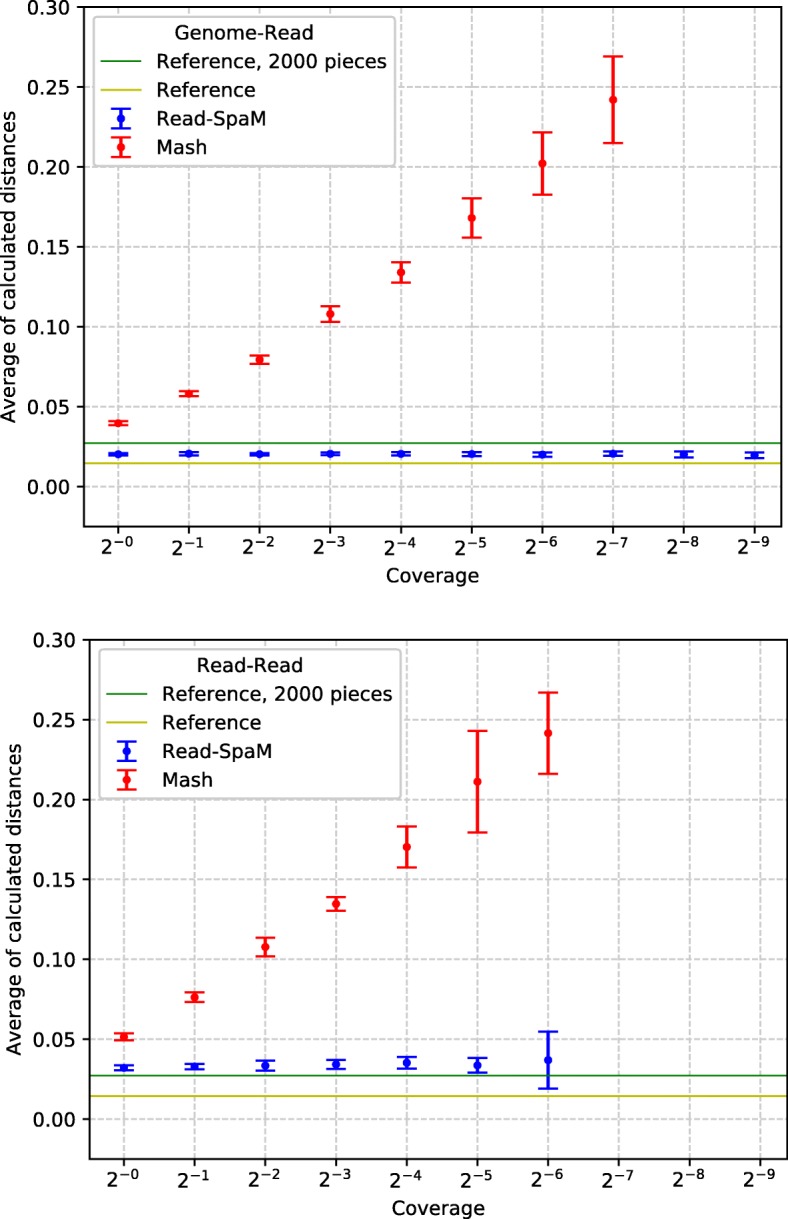
Estimated distances between *E.coli* strains IAI1 and F2a2457T as in Fig. 7, using unassembled reads from one genome and the assembled second genome (top) and unassembled reads from both genomes (bottom)

The results of *Read-SpaM* and *Mash* on two pairs of real genomes from *E. coli* are shown in Figs. 7 and 8. As a comparison, the distances calculated by *FSWM* on the whole genomes and on the fragmented genomes, as explained above, are shown as horizontal lines. As in the previous tests, we compared assembled genomes to sets of simulated reads and sets of reads from both genomes. Again, these figures show the average distances and standard deviations over 10 sets of simulated reads for each level of sequencing coverage. As above, these average values are shown only if distances could be estimated for *all* of the 10 sets of reads.

**Fig. 9:**
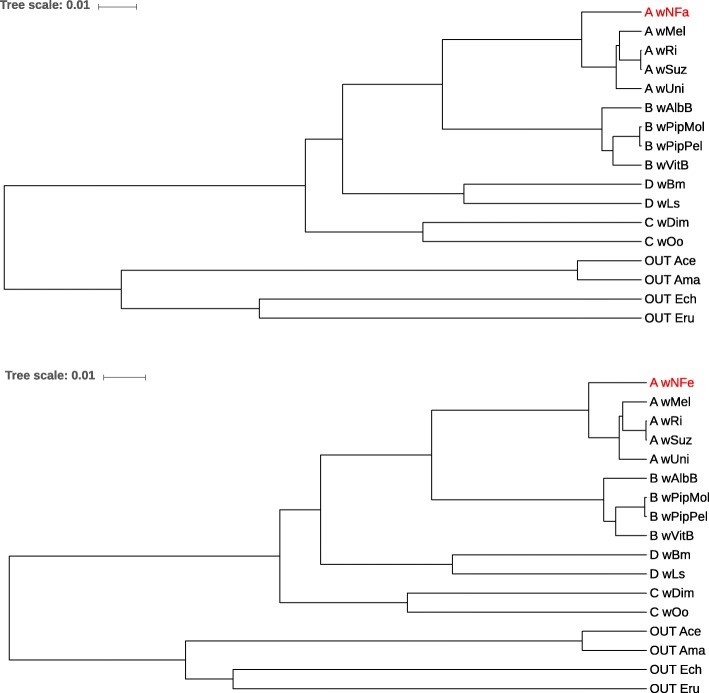
Phylogenetic trees for a set of 13 *Wolbachia* strains from super groups *A*−*D* plus 4 strains from the closely related alphaproteobacterial genera *Anaplasma* and *Ehrlichia* as outgroup. For each tree, we used the full genome sequences from 12 *Wolbachia* strains and the outgroup strains. For the 13th *Wolbachia* strain, we used sets of unassembled sequencing reads with coverage 1*X*. The strain with the unassembled reads was *wNFa* (top) and *wNFe* (bottom)

**Fig. 10:**
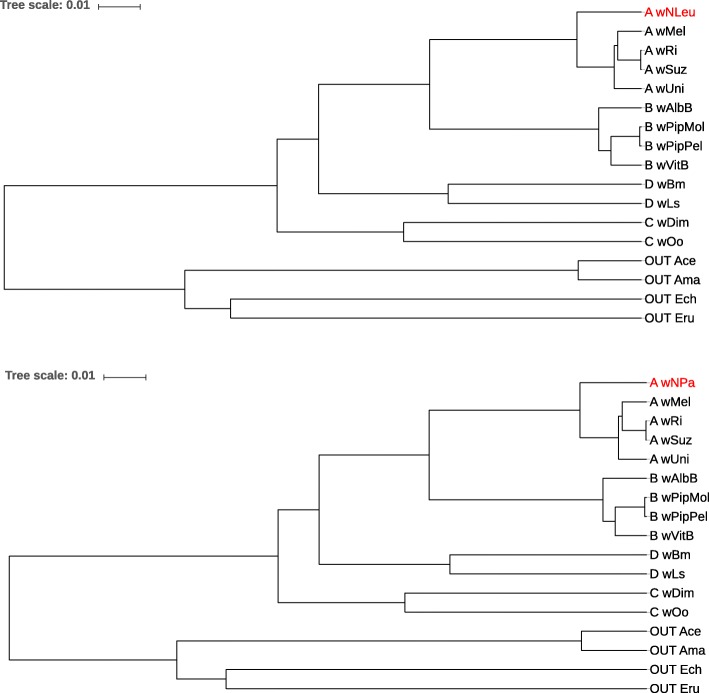
Phylogenetic trees for 17 bacterial strains as in Fig. 9. Here, we used unassembled reads from strains *wNLeu* (top) and *wNPa* (bottom) as input sequences, for the respective other strains we used their full genome sequences

Finally, Fig. 9 and Fig. 10 show phylogenetic trees reconstructed from 13 *Wolbachia* genomes plus 4 outgroup genomes. For each tree, unassembled reads from one *Wolbachia* genome were used with sequencing coverage 1*X* (shown in red in the figures), together with the assembled genomes from the remaining 16 taxa. The topologies of the trees that we obtained is exactly the same as for the reference tree from[50]. We also did the same test runs with lower sequencing coverage and obtained the same correct topologies.

As mentioned above, we had to adjust the length of the patterns and spaced-word matches, respectively, in *Read-SpaM*, compared to the original version of *FSWM*. To find a suitable pattern length, we evaluated patterns with length between 52 and 120. If the patterns were too long, only few spaced-word matches were found, especially for low sequencing coverage and for sequence pairs with a low degree of similarity. This led to statistically unstable distance estimates. If the patterns were too short, on the other hand, we found that the accuracy of the estimated distances decreased. We found that a pattern length of 72 worked best for *Read-SpaM* on our semi-artificial genome sequences, so we are using this value as the default pattern length.

### Runtime

Table 1 shows the the runtimes of *Read-SpaM* and *Mash* for comparing two strains of *E. coli*. For *Read-SpaM*, the runtime is between 0.8 s and 3.4 s, depending on the level of sequencing coverage. As a comparison, a run of *FSWM* on the corresponding assembled genomes takes around 6 s.

**Table 1:** Runtime of *Read-SpaM* and *Mash* (in seconds) to estimate the distance between two strains of *E. coli*, by comparing an assembled genome to unassembled reads and by comparing unassembled reads from both strains to each other, for varying levels of sequencing coverage

Coverage	Genome vs. Read		Read vs. Read	
	*Multi-SpaM*	*Mash*	*Multi-SpaM*	*Mash*
1*X*	3.40	0.35	2.84	0.35
2^−1^*X*	2.43	0.27	1.55	0.19
2^−2^*X*	2.02	0.24	1.16	0.11
2^−3^*X*	1.86	0.22	1.00	0.06
2^−4^*X*	1.74	0.19	0.93	0.04
2^−5^*X*	1.76	0.20	0.89	0.02
2^−6^*X*	1.64	0.20	0.87	0.01
2^−7^*X*	1.64	0.22	0.86	0.01
2^−8^*X*	1.66	0.19	0.87	0.00
2^−9^*X*	1.63	0.19	0.85	0.02

## Discussion

In this paper, we introduced *Read-SpaM*, an adaption of our previously published software *Filtered Spaced Word Matches (FSWM)* to estimate phylogenetic distances based on unassembled sequencing reads. We evaluated this approach on real and semi-artificial bacterial genomes with varying phylogenetic distances and for varying levels of sequencing coverage.

Figure 2 shows that, if unassembled reads from one bacterium are compared to an assembled genome from a second bacterium, distances predicted by *Read-SpaM* are fairly accurate, even for very low levels of sequencing coverage. For sequencing coverage down to 2^−7^*X*, *Read-SpaM* produced good results for the whole range of distances that we tested, i.e. for up to 1 substitution per position under the *Jukes-Cantor* model. For a coverage of 2^−8^*X* and 2^−9^*X*, our program still returned good results, but only for distances up to 0.8 substitutions per position. For larger distances it happened, at this low level of sequencing coverage, that no spaced-word matches between the sequences were found, so no results could be produced any more.

As can be expected, the range of sequencing coverage and evolutionary distances where *Read-SpaM* works reliably, is smaller if unassembled reads from *both* genomes are used as input. As shown in Fig 4, in this situation distances can be estimated only for a sequencing coverage down to 2^−6^*X*. For this coverage, distances up to 0.4 substitutions per position can still be estimated, but for lower levels of sequencing coverage, no meaningful results are produced, since not enough spaced-word matches can be found any more.

Our results also show that, in general, *Read-SpaM* tends to over-estimate phylogenetic distances somewhat, especially for low levels of sequencing coverage. A possible explanation is that, for short sequencing reads and low levels of coverage, only relatively few spaced-word matches can be found that represent true homologies. With the cut-off value that we are using to distinguish between homologous and background spaced-word matches, it is always possible that some random spaced-word matches have scores above our threshold. In situations where only a small number of homologous spaced-word matches is found, these background matches can lead to over-estimated distances.

On the pairs of real-world *E. coli* genomes, distances estimated by *Read-SpaM* were again larger than the reference distances that we calculated, in this case, with *FSWM*, applied to the assembled genomes. Here, there may be another reason for this discrepancy, in addition to the above mentioned over-estimation of distances by *Read-SpaM* caused by random spaced-word matches. As explained in “Real-world genome pairs” section, *FSWM* often under-estimates distances between real-world genomes, since most spaced-word matches are found in regions of high sequence similarity, so these regions dominate the distance estimates. It is therefore possible that the *Read-SpaM* distances are *more* accurate than the ones estimated by *FSMW*. In Figs. 7 and Fig. 8, we also used ‘corrected’ *FSWM* distances, obtained by splitting one of the compared genomes into fragments, see above. It should be clear that this is only a very rough way of mitigating the bias in *FSWM*. The ‘reference distances’ in these figures can, thus, only be seen as rough approximations to the real distance between the genomes, to obtain reliable reference distances, one would need alignments of the compared genome sequences.

In our test runs with reads from real-world genome sequences, we observed a similar result as with our semi-artificial sequences. If simulated reads from *both* compared genomes are used then, for very low levels of sequencing coverage, the estimated distances become not only more noisy, as one would expect, but they also become larger, compared to the test runs with higher sequencing coverage. Again, the over-estimation of phylogenetic distances may be due to the fact that only few *homologous* spaced-word matches are found if the coverage becomes low, so spurious random spaced-word matches with scores slightly about the threshold, may influence the estimated distances. Experiments with varying threshold values may help to clarify this point.

Additional benchmark results for *Read-SpaM* can be found in the recently published *AFproject* study [9]. Here, a large number of alignment-free methods were evaluated and compared to each other on various test data sets.

In our program evaluation, we also ran the program *Mash* [24] on the same data sets. *Mash* is a widely used and extremely fast program that can accurately estimate phylogenetic distances between DNA sequences based on their *k*-mer content. In our study, we could confirm that this program can accurately estimate distances between unassembled reads and assembled genomes. The range of sequencing coverage and evolutionary distances, however, where *Mash* can be applied with its default parameter values is considerably smaller than for *Read-SpaM*, as can be seen in Fig 2 to Fig 5. Even within this range, the distance estimates by *Mash* seem to be less accurate, in general, than the estimates by *Read-SpaM*. If sets of reads are compared to each other, *Mash* substantially over-estimates phylogenetic distances, especially if the sequencing coverage is low.

The relative inaccuracy of *Mash* on sets of reads with low coverage can be explained by way in which this program estimates distances. *Mash* calculates the *Jaccard index* of the *k*-mer sets of the compared genomes [41, 53], i.e. it compares the number of *k*-mers that are found in both genomes simultaneously to the total number of *k*-mers in the genomes. In other words, it compares the number of *k*-mer matches to the *length* of the compared genomes. This is a very efficient and accurate way of estimating the number of mismatches in the (unknown) alignment of the two genomes, and thereby their phylogenetic distance.

On the downside, this approach has to assume that the compared genomes are related to each other over their entire length. As the authors of *Mash* put it, the *Jaccard index is a useful measure of global sequence similarity* but is *sensitive to genome size* [24]. As a consequence, *Mash* overestimates phylogenetic distances if the compared sequences share only *local* homologies [27]. This is the case if we compare a set of reads with low sequencing coverage to an assembled genome, or two sets of reads to each other. It may be possible to obtain results with *Mash* on reads with a lower coverage by adapting the program parameters accordingly. If the *sketch* size would be increased and the *k*-mer length reduced, *Mash* might produce distance values for data sets where it did not produce meaningful output with default values. A systematic evaluation of different parameter settings in *Mash* was, however, beyond the scope of the present study. An alternative to *Mash* could be the recently developed program *Skmer* [37] which also works on unassembled reads and which has been designed to deal with low sequencing coverage.

While, on our test data, *Read-SpaM* produced more accurate phylogenetic distances than *Mash* and was applicable to more distantly related genomes with much lower sequencing coverage, an important advantage of *Mash* is its high speed. Table 1 shows that, on most test data, *Mash* is roughly one order of magnitude faster than *Read-SpaM*. This is due to the fact that *Mash* is based on *k*-mer counting, while *Read-SpaM* evaluates the number of mismatches for every space-word match with respect to the specified pattern *P*. As expected, read-read comparison is faster than genome-read comparison for both of the evaluated programs, for all levels of sequencing coverage. For both methods, the runtime decreases heavily in the beginning but only small differences can be found for a coverage below around 2^−4^*X*.

## Conclusion

Our program evaluation shows that read-based estimation of phylogenetic distances with *Read-SpaM* has a high potential. The developed approach should be particularly useful for phylogenetic distances below 0.6 substitutions per position, and if unassembled reads are to be compared to assembled genomes. An important application is, for example, to search for the position of a previously unknown species in an existing phylogenetic tree, the so-called *phylogenetic placement* problem [54–59]. In this situation, low-pass sequencing can be an attractive alternative to phylogenetic barcoding based on selected *marker genes* [60, 61] to identify the phylogenetic position of an unknown species. As read-to-read comparison with *Read-SpaM* still produces reliable results for sequencing coverage down to 2^−3^*X*, it is possible to estimate phylogenetic distances between strains or species for which assembled genomes are not available.

## Data Availability

Our software is freely available at: https://github.com/burkhard-morgenstern/Read-SpaM

## Abbreviations

bp: base pair
FSWM: Filtered Spaced Word Matches
